# Effect of interval between neoadjuvant chemoradiotherapy and surgery on oncological outcomes in poor responders with locally advanced rectal cancer: a retrospective cohort study

**DOI:** 10.1097/JS9.0000000000000438

**Published:** 2023-05-13

**Authors:** Dakui Luo, Yufei Yang, Ruijia Zhang, Qingguo Li, Xinxiang Li

**Affiliations:** a Department of Colorectal Surgery, Fudan University Shanghai Cancer Center; b Department of Oncology, Shanghai Medical College, Fudan University, Shanghai, China

**Keywords:** locally advanced rectal cancer, neoadjuvant chemoradiation, poor responders, surgical interval

## Abstract

The optimal interval from completion of neoadjuvant chemoradiotherapy (CRT) to surgery in locally advanced rectal cancer remains controversial. It seems that delayed surgery is associated with an increase in pathological complete response rates. However, the prognostic effect of delayed surgery in poor responders is unclear.

**Materials and methods:**

Patients with locally advanced mid or distal rectal cancer undergoing neoadjuvant CRT followed by total mesorectal excision at a university teaching cancer center between June 2010 and December 2018 were retrospectively reviewed in this study. According to the tumor regression grade, poor responders (tumor regression grade 2–3) to neoadjuvant CRT were selected for analyses. Patients were divided into the longer interval group (greater than 8 weeks) and the shorter interval group (8 weeks or less) based on the wait time from completion of neoadjuvant CRT therapy to surgery. Results: among 916 eligible patients, 522 patients had a poor tumor response. There were 217 patients in the shorter interval group and 305 patients in the longer interval group. At the baseline, patients in the longer interval group were more likely to have a T3 stage and positive vascular invasion. Compared with patients in the shorter interval group, patients in the longer interval group had significantly worse overall survival and disease-free survival (DFS) (log-rank test, overall survival: *P*=0.004, DFS: *P*<0.001). The 3-year DFS rates were 75.6 and 63.1% in the shorter interval group and the longer interval group, respectively. In the multivariate analysis, delayed surgery was associated with an increased risk of mortality (hazard ratio: 2.003, 95% CI: 1.233–3.253, *P*=0.005) and recurrence (hazard ratio: 1.555, 95% CI: 1.121–2.156, *P*=0.008).

**Conclusion:**

Patients who had a poor tumor response should be identified by restaging MRI and receive radical surgery in time, without a prolonged interval.

## Introduction

Highlights

- This is a large cohort study to discuss the prognostic effect of a delayed surgery in patients who had a poor tumor response after completion of neoadjuvant chemoradiotherapy for locally advanced rectal cancer based on tumor regression grade.
- Patients who had a poor tumor response should be identified by restaging MRI in time and received radical surgery without a prolonged interval.

Neoadjuvant chemoradiotherapy (CRT) followed by total mesorectal excision (TME) and adjuvant chemotherapy has been recommended as the standard therapy for locally advanced rectal cancer (LARC). Compared with postoperative CRT, neoadjuvant CRT had the advantages of improved resectability and local control, as well as less therapeutic toxicity^1,2^. Our previous study demonstrated that preoperative radiotherapy was superior to postoperative radiotherapy in terms of oncological outcomes in metastatic rectal cancer patients who received definitive surgical resection of the primary tumor^3^.

After receiving neoadjuvant CRT, patients showed varying degrees of tumor downsizing and downstaging. Approximately 20% of patients achieved a pathological complete response (pCR), with excellent long-term outcomes^4–6^. This subgroup has the potential of receiving a ‘watch-and-wait’ approach without radical surgery^7^.

Several tumor regression grading (TRG) systems have been developed to stratify tumor pathological response to neoadjuvant therapy^8,9^. To date, there is no uniform consensus on whether TRG is a prognostic factor in LARC^10–13^.

Coordination of neoadjuvant CRT and surgery is also important because delayed surgery is associated with higher pCR rates^14–16^. Recently, a pooled analysis of 3085 patients from seven randomized trials suggested that the best time to achieve pCR in LARC is at 10 weeks and lengthening the surgical interval had no impact on oncological outcomes^17^. However, several studies have demonstrated that a longer interval is associated with higher rates of positive margins and lower rates of sphincter preservation^18,19^. It is rational to prolong the surgical interval to achieve maximum tumor regression in patients who eventually have a complete or good response confirmed by pathology. Nevertheless, it is unclear whether delayed surgery has a clinical benefit in poor responders.

The aim of this study was to determine the prognostic implications of the surgical interval after neoadjuvant CRT in LARC patients who had a poor tumor response based on the TRG score.

## Materials and methods

### Study population

The records of rectal cancer patients who received surgery at a university teaching cancer center between June 2010 and December 2018 were retrospectively reviewed. A biopsy was performed on all patients to obtain histological confirmation. Patients were staged before neoadjuvant CRT using computed tomography (CT) scans as well as MRI. The inclusion criteria were as follows: patients were diagnosed with LARC, patients received neoadjuvant CRT and curative surgery, mid to low rectal adenocarcinoma was diagnosed by endoscopy (defined as the distal edge of the tumor within 10 cm from the anal verge), pathologically confirmed TRG2–3. The exclusion criteria were as follows: patients received neoadjuvant chemotherapy alone, patients were diagnosed with stage IV disease, and patients received palliative surgery. The following characteristics were extracted: TRG score; age at diagnosis; sex; histologic type; differentiation; yield pathological T stage; ypN stage; perineural invasion; vascular invasion; surgical procedures; and survival data. The study protocol was approved by the ethics committee of the university teaching cancer center. This work was reported in line with the strengthening the reporting of cohort studies in surgery (STROCSS) criteria^20^, (Supplemental Digital Content 1, http://links.lww.com/JS9/A489).

### Neoadjuvant CRT and surgical resection

In general, patients received pelvic radiation at a dose of 50 Gy/25 fractions with concurrent capecitabine at 825 mg/m^2^ twice daily for 5 days/week. Consolidation chemotherapy with one or more cycles of capecitabine plus oxaliplatin was performed 2 weeks after the end of CRT (oxaliplatin 130 mg/m^2^ on day 1 and capecitabine 1000 mg/m^2^ twice daily on days 1–14). The detailed cycles of consolidation chemotherapy depended on the surgical interval. The surgical interval was defined as the waiting time from the completion of CRT to surgery. Surgical resection was performed at least four weeks after the completion of neoadjuvant CRT. All patients received TME. In general, a total of six cycles of chemotherapy (preoperative and postoperative) was administered.

### TRG score

The TRG was scored by two independent specialist pathologists who were not aware of the MRI diagnosis according to the seventh edition of the American Joint Committee on Cancer Staging Manual and the College of American Pathologists Guidelines as modified by Ryan *et al*.^21^. The 4-point TRG system was graded on a scale of 0–3. TRG0: complete regression with no residual cancer cells; TRG1: almost complete regression with only one single residual cancer cell or a cluster of cancer cells; TRG2: moderate regression with many residual cancer cells; TRG3: minimal regression with nearly no cancer cells killed. TRG scoring was evaluated in the pathological report. In the present study, patients with TRG2–3 were considered poor responders.

### Follow-up

Clinical examination, tumor marker assessment, colonoscopy, chest and abdominal CT scan, and pelvic CT or MRI were included in the follow-up protocol. Medical records review, telephonic follow-ups, and death registry data linkage were used in combination for collecting survival data. The last follow-up was on 30 September 2020. The primary endpoints were overall survival (OS) and disease-free survival (DFS). OS was defined as the time from the initiation of therapy to the final follow-up or until death from any cause. DFS was defined as the time from the initiation of therapy to the date of local or distant relapse, death from any cause or the date of last follow-up.

### Statistical analysis

All statistical analyses were conducted using SPSS version 25.0 (SPSS). *χ* ^2^-test was used to compare categorical variables between two groups. Survival analyses were undertaken using Kaplan–Meier analysis. The log-rank test was used for univariate comparisons. Multivariate Cox regression analysis was performed on variables that were significant in univariate analysis to identify the independent prognostic factors. A *P*<0.05 was considered as statistically significant.

## Results

A total of 7632 rectal cancer patients were retrospectively reviewed. Of these, 916 patients with locally advanced (clinical stage II or III) mid or distal rectal cancer underwent neoadjuvant CRT followed by TME. Finally, a total of 522 patients who had a poor tumor response were included in the analysis. The detailed patient selection process is shown in Supplementary Figure 1. (Supplemental Digital Content 2, http://links.lww.com/JS9/A490). Patients were divided into the longer interval group (greater than 8 weeks) and the shorter interval group (8 weeks or less) based on the wait time from completion of neoadjuvant CRT therapy to surgery. There were 217 patients (41.6%) in the shorter interval group and 305 patients (58.4%) in the longer interval group. The median surgical interval was 7.1 weeks for the shorter interval group and 10.0 weeks for the longer interval group.

Patient demographics are summarized in Table 1. Patients in the longer interval group were more likely to have a T3 stage and positive vascular invasion. The other characteristics were comparable between the two groups.

**Table 1:** Demographical characteristics of patients in this study.

		Interval		
Characteristic	Total (*N*=522)	≤8 weeks (*N*=217)	>8 weeks (*N*=305)	*P*
Age (years)				0.685
≤50	142	57 (40.1)	85 (59.9)	
>50	380	160 (42.1)	220 (57.9)	
Sex				0.114
Male	345	135 (39.1)	210 (60.9)	
Female	177	82 (46.3)	95 (53.7)	
Histologic type				0.094
Adenocarcinoma	484	207 (42.8)	277 (57.2)	
Mucinous	30	9 (30.0)	21 (70.0)	
Signet ring cell	8	1 (12.5)	7 (87.5)	
Differentiation				0.052
Poor	91	27 (29.7)	64 (70.3)	
Moderate	372	160 (43.0)	212 (57.0)	
Well	2	1 (50.0)	1 (50.0)	
Unknown	57	29 (50.9)	28 (49.1)	
ypT stage				0.003
T1	16	9 (56.3)	7 (43.8)	
T2	121	58 (47.9)	63 (52.1)	
T3	319	112 (35.1)	207 (64.9)	
T4	51	29 (56.9)	22 (43.1)	
Unknown	15	9 (60.0)	6 (40.0)	
ypN stage				0.548
N0	289	114 (39.4)	175 (60.6)	
N1	181	80 (44.2)	101 (55.8)	
N2	52	23 (44.2)	29 (55.8)	
Perineural invasion				0.702
Negative	390	164 (42.1)	226 (57.9)	
Positive	132	53 (40.2)	79 (59.8)	
Vascular invasion				0.005
Negative	453	199 (43.9)	254 (56.1)	
Positive	69	18 (26.1)	51 (73.9)	
Surgical procedures				0.229
APR	197	79 (40.1)	118 (59.9)	
AR	275	122 (44.4)	153 (55.6)	
Hartmann	50	16 (32.0)	34 (68.0)	
TRG				0.108
2	418	181 (43.3)	237 (56.7)	
3	104	36 (34.6)	68 (65.4)	

**Figure 1:**
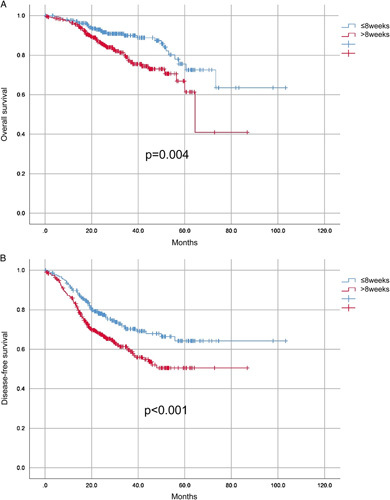
Survival curve of poor responders with locally advanced rectal cancer treated with neoadjuvant chemoradiotherapy and total mesorectal excision. Kaplan–Meier analysis of (A) overall survival and (B) disease-free survival in patients stratified by different surgical intervals (8 weeks or less vs. greater than 8 weeks).

The median follow-up was 30.8 months. During the follow-up, 170 patients (32.6%) experienced a recurrence and 86 patients (16.5%) died. Patients in the longer interval group had significantly worse OS (log-rank test, *P*=0.004) and DFS (log-rank test, *P*<0.001) as compared to patients in the shorter interval group (Fig. 1). The 3-year DFS rates were 75.6 and 63.1% in the shorter interval group and the longer interval group, respectively. Multivariate Cox analysis revealed that delayed surgery was associated with an increased risk of mortality (hazard ratio: 2.003, 95% CI: 1.233–3.253, *P*=0.005) (Table 2) and recurrence (hazard ratio: 1.555, 95% CI: 1.121–2.156, *P*=0.008) (Table 3). The rates of local-recurrence were 6.9% (15/217) and 9.8% (30/305) in the shorter interval group and the longer interval group, respectively. Patients in the shorter interval group showed marginal evidence of better local-recurrence free survival as compared to patients in the longer interval group (log-rank test, *P*=0.073) (Supplementary Figure 2. Supplemental Digital Content 3, http://links.lww.com/JS9/A491).

**Table 2:** Univariate and multivariate analyses of the factors for overall survival.

	Univariate analysis		Multivariate analysis		
Variable	HR (95% CI)	*P*	HR (95% CI)	*P*
Age (years)				
≤50	Reference		NI	
>50	1.195 (0.731–1.955)	0.477		
Sex				
Male	Reference		NI	
Female	1.059 (0.684–1.641)	0.797		
Histologic type				
Adenocarcinoma	Reference		NI	
Mucinous	1.226 (0.446–3.371)	0.693		
Signet ring cell	8.148 (2.927–22.680)	<0.001		
Unknown	/			
Differentiation				
Poor	Reference		Reference	
Moderate	0.288 (0.181–0.459)	<0.001	0.448 (0.274–0.734)	0.001
Well	/		/	
Unknown	/		/	
ypT stage				
T1	Reference		NI	
T2	0.805 (0.176–3.684)	0.779		
T3	2.173 (0.527–8.955)	0.283		
T4	1.833 (0.417–8.061)	0.423		
Unknown	/		/	
ypN stage				
N0	Reference		Reference	
N1	1.900 (1.178–3.063)	0.008	1.381 (0.834–2.285)	0.209
N2	4.122 (2.304–7.376)	<0.001	3.415 (1.773–6.576)	<0.001
Perineural invasion				
Negative	Reference		Reference	
Positive	2.868 (1.866–4.410)	<0.001	2.119 (1.309–3.428)	0.002
Vascular invasion				
Negative	Reference		Reference	
Positive	2.690 (1.586–4.563)	<0.001	1.047 (0.562–1.952)	0.885
Surgical procedures				
APR	Reference		Reference	
AR	0.495 (0.302–0.810)	0.005	0.579 (0.352–0.953)	0.032
Hartmann	2.916 (1.679–5.064)	<0.001	3.337 (1.893–5.880)	<0.001
TRG				
2	Reference		NI	
3	1.413 (0.881–2.268)	0.152		
Interval				
≤8 wk	Reference		Reference	
>8 wk	1.940 (1.224–3.075)	0.005	2.003 (1.233–3.253)	0.005

**Table 3:** Univariate and multivariate analyses of the factors for disease-free survival.

	Univariate analysis		Multivariate analysis	
Variable	HR (95% CI)	*P*	HR (95% CI)	*P*
Age (years)				
≤50	Reference		NI	
>50	1.044 (0.742–1.468)	0.806		
Sex				
Male	Reference		NI	
Female	0.948 (0.690–1.302)	0.741		
Histologic type				
Adenocarcinoma	Reference		NI	
Mucinous	1.046 (0.534–2.052)	0.895		
Signet ring cell	2.552 (0.943–6.906)	0.065		
Unknown	/			
Differentiation				
Poor	Reference		Reference	
Moderate	0.484 (0.341–0.688)	<0.001	0.668 (0.459–0.973)	0.035
Well	/		/	
Unknown	/		/	
ypT stage				
T1	Reference		NI	
T2	0.816 (0.282–2.360)	0.707		
T3	1.937 (0.715–5.251)	0.194		
T4	1.404 (0.477–4.129)	0.538		
Unknown	/		/	
ypN stage				
N0	Reference		Reference	
N1	1.372 (0.988–1.905)	0.059	1.137 (0.805–1.607)	0.466
N2	2.336 (1.499–3.641)	<0.001	1.778 (1.084–2.919)	0.023
Perineural invasion				
Negative	Reference		Reference	
Positive	2.430 (1.785–3.309)	<0.001	2.004 (1.427–2.814)	<0.001
Vascular invasion				
Negative	Reference		Reference	
Positive	2.086 (1.410–3.086)	<0.001	1.099 (0.696–1.733)	0.686
Surgical procedures				
APR	Reference		Reference	
AR	0.607 (0.437–0.843)	0.003	0.639 (0.458–0.892)	0.008
Hartmann	1.837 (1.183–2.852)	0.007	1.849 (1.187–2.880)	0.007
TRG				
2	Reference		NI	
3	1.342 (0.947–1.902)	0.098		
Interval				
≤8 weeks	Reference		Reference	
>8 weeks	1.606 (1.168–2.207)	0.004	1.555 (1.121–2.156)	0.008

Besides that, tumor differentiation, ypN stage, vascular invasion, and surgical procedures were also found to be independently associated with OS and DFS in poor responders.

**Figure 2:**
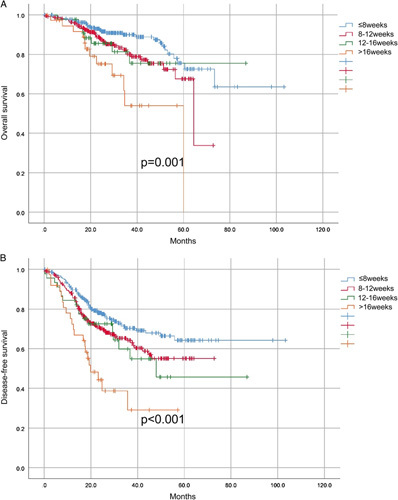
Survival curve of indicated patients. Kaplan–Meier analysis of (A) overall survival and (B) disease-free survival in patients stratified for every 4 weeks of waiting time after 8 weeks.

Furthermore, patients had a trend of lower survival as waiting time was prolonged, especially for more than 16 weeks (Fig. 2).

## Discussion

The optimal time interval between neoadjuvant CRT and surgery in LARC patients remains unknown. Special attention was paid to the correlation between a longer waiting period and an improvement in the rate of pCR since pCR is considered as a surrogate marker for estimating long-term survival benefits. Notably, a watch-and-wait strategy is a valuable alternative for rectal resection in patients who were evaluated with a clinical complete response. Patients could undergo salvage surgical treatment for local regrowth with adequate pelvic control^22^. To achieve enhanced tumor regression, total neoadjuvant therapy (TNT), defined as delivering all planned chemotherapy before surgery, was developed. A higher pCR rate in a TNT approach might result in more patients adopting a watch-and-wait strategy^23^, which can be considered as the goal toward delaying surgery indefinitely until the tumor regrows. To date, multiple trials have compared different TNT strategies with standard CRT therapy. Yet, the oncological safety of TNT remains controversial. Particularly, the updated RAPIDO results demonstrated that a TNT strategy (short-course radiotherapy, chemotherapy, and surgery) was associated with an increased risk of locoregional recurrence and the reduction in disease-related treatment failure and distant metastases compared with the standard-care group^24^. Notably, a meta-analysis conducted sensitivity analyses and concluded that the decision to make adjuvant chemotherapy optional in the standard-care group may have biased the results in favor of the above TNT strategy^25^.

Evidence indicated that a longer waiting period after neoadjuvant CRT does not improve the oncological outcomes of LARC^26^, which raises the question whether the survival benefit of patients achieving a satisfactory pathological response is impaired or masked by poor responders. Patients with a pCR are likely to have better outcomes, but improved DFS or OS is not observed in the entire population. Therefore, some patients with intensification may have a worse outcome, and resistant tumors may become even more resistant and aggressive. Moreover, delayed surgery was associated with increased complications and a worse quality of mesorectal resection^27^. To the best of our knowledge, this is the first study to investigate the prognostic implication of the surgical interval after neoadjuvant CRT in LARC patients who had a poor tumor response based on the TRG score. This study showed that poor responders in the longer interval group had significantly worse OS and DFS compared with those in the shorter interval group. In this study, the therapy was not suspended in the longer interval group. Consolidation chemotherapy was performed 2 weeks after the end of CRT until the surgery was scheduled. Therefore, the rationality of adopting the TNT strategy in patients who had a poor tumor response remains unclear. For good responders, a longer waiting time may be suitable to achieve a clinical response. A prospective observational study with weekly MRI found that tumor shrinkage is the fastest in the beginning of treatment (26%/week), slows to 7%/week in the last 2 weeks of CRT, and finally to 1.3%/week in the last 5 weeks before surgery^28^. It is difficult to achieve a pCR for some patients with TRG2/TRG3 who received delayed surgery. Therefore, poor responders could be identified by restaging MRI in the early stages and receive radical surgery timely. TNT seems more appropriate for good responders during the CRT course itself.

Recently, a multicenter retrospective cohort study reported that a longer interval before surgery after completing neoadjuvant CRT was associated with worse oncological outcomes in tumors with a poor pathological response to neoadjuvant CRT^29^. Our results are consistent with this conclusion. Nevertheless, they defined a ypT stage of 2 to 3 or a ypN positive as a minor or poor response from the point of yp stage instead of the TRG score. Downstaging cannot accurately reflect the tumor response to neoadjuvant therapy. In particular, if patients achieve a good response with only microscopic foci of tumor cells in the subserosa, they would be staged as ypT3. Besides that, there were more deaths (37.7%) than recurrences (26.9%) in this study, indicating that several patients may die from noncancer-related causes.

Notably, patients with a poor tumor response need to be identified in time and receive radical surgery without delay. How to accurately identify the poor responders in time? Preoperative response evaluation with neoadjuvant CRT remains a challenge in LARC. MRI is accurate and sensitive to evaluate the tumor response to neoadjuvant CRT. In previous studies, the MRI-TRG system predicted survival outcomes for the favorable and poor responders^30,31^. Sclafani *et al.* ^32^ explored the correlation between MRI tumor regression grade (mrTRG) and pathological tumor regression grade (pTRG) in rectal cancer, and concluded that the agreement between mrTRG and pTRG is low and mrTRG cannot be used as a surrogate of pTRG. Pang *et al.* proposed a new four-category MRI-TRG system based on the volumetric analysis of the residual tumor and CRT-induced anorectal fibrosis, which could be a surrogate for the TRG classification scheme^33^. Furthermore, radiomics of pretreatment MRIs and a deep learning model based on diffusion kurtosis MRI could show good performance for predicting tumor response^34,35^.

This study had some limitations. First, this retrospective study may have introduced potential bias and confounding variables. For example, there were more patients with mucinous and Signet ring cell histology in the long interval group. Second, data on several important clinicopathological characteristics, such as detailed clinical stages, cycles of preoperative chemotherapy, MSI status, *et al.*, were not recorded. Third, circumferential resection margin (CRM) status is a very strong independent predictor of survival; however, the effects of CRM status on oncological outcomes were not evaluated due to the limited number of patients with positive CRM. Fourth, it is more difficult to assess clinical response with MRI or CT in large, circumferential or mucinous/signet ring tumors, which requires time to determine whether or not the tumors respond to treatment.

## Conclusion

In summary, poor responders should be identified by restaging MRI and receive radical surgery in time, without a delay.

## Ethics approval and consent to participate

This study was approved by the Institutional Review Board of Fudan University Shanghai Cancer Center. The written informed consent was obtained from all patients or legally authorized representative of dead participants.

## Sources of funding

This work was supported by the National Natural Science Foundation of China (Grant No. 81972260; No. 82103259), Shanghai Municipal Natural Science Foundation (21ZR1414400), and Shanghai Medical Innovation Research Project (22Y11907600). The funders had no role in the study design, data collection and analysis, decision to publish, or manuscript preparation.

## Author contribution

D.L.: conceptualization, data curation, methodology, software, writing – original draft; Y.Y.: conceptualization, data curation, methodology, writing – original draft; R.Z.: writing – review and editing; Q.L.: writing – review and editing, supervision; X.L.: conceptualization, methodology, supervision, writing – review and editing.

## Conflicts of interest disclosure

The authors declare that the research was conducted in the absence of any commercial or financial relationships that could be construed as a potential conflict of interest.

## Research registration unique identifying number (UIN)

1. Research Registry. 2. Name of the registry: Re-evaluating the prognostic value of tumor regression grade in patients with rectal cancer after preoperative chemoradiotherapy: A High-Volume Center Retrospective Analysis. 3. UIN: researchregistry 7426. 4. www.researchregistry.com/register now#home/registrationdetails/61aa2261474a930020ea8b1d/.

## Guarantor

Xinxiang Li.

## Data availability statement

The FUSCC dataset used during the current study are available from the corresponding author on reasonable request.

## Provenance and peer review

Not commissioned, externally peer-reviewed.

## Supplementary Material

## References

[1] Sauer R Becker H Hohenberger W . Preoperative versus postoperative chemoradiotherapy for rectal cancer. N Engl J Med 2004;351:1731–1740. 15496622 10.1056/NEJMoa040694

[2] Sauer R Liersch T Merkel S . Preoperative versus postoperative chemoradiotherapy for locally advanced rectal cancer: results of the German CAO/ARO/AIO-94 randomized phase III trial after a median follow-up of 11 years. J Clin Oncol 2012;30:1926–1933. 22529255 10.1200/JCO.2011.40.1836

[3] Luo D Liu Q Zhu J . Survival benefit of preoperative versus postoperative radiotherapy in metastatic rectal cancer treated with definitive surgical resection of primary tumor: a population based, propensity score-matched study. J Cancer 2019;10:1307–12. 30854140 10.7150/jca.28320 PMC6400684

[4] Bosset JF Calais G Mineur L . Fluorouracil-based adjuvant chemotherapy after preoperative chemoradiotherapy in rectal cancer: long-term results of the EORTC 22921 randomised study. Lancet Oncol 2014;15:184–190. 24440473 10.1016/S1470-2045(13)70599-0

[5] Das P Skibber JM Rodriguez-Bigas MA . Predictors of tumor response and downstaging in patients who receive preoperative chemoradiation for rectal cancer. Cancer 2007;109:1750–1755. 17387743 10.1002/cncr.22625

[6] Park IJ You YN Agarwal A . Neoadjuvant treatment response as an early response indicator for patients with rectal cancer. J Clin Oncol 2012;30:1770–1776. 22493423 10.1200/JCO.2011.39.7901 PMC3383178

[7] Glynne-Jones R Hughes R . Critical appraisal of the ‘wait and see’ approach in rectal cancer for clinical complete responders after chemoradiation. Br J Surg 2012;99:897–909. 22539154 10.1002/bjs.8732

[8] Kim SH Chang HJ Kim DY . What is the ideal tumor regression grading system in rectal cancer patients after preoperative chemoradiotherapy? Cancer Res Treat 2016;48:998–1009. 26511803 10.4143/crt.2015.254 PMC4946373

[9] Germani P Di Candido F Leonard D . Contemporary snapshot of tumor regression grade (TRG) distribution in locally advanced rectal cancer: a cross sectional multicentric experience. Updates Surg 2021;73:1795–803. 33818750 10.1007/s13304-021-01044-0 PMC8500860

[10] Abdul-Jalil KI Sheehan KM Kehoe J . The prognostic value of tumour regression grade following neoadjuvant chemoradiation therapy for rectal cancer. Colorectal Dis 2014;16:O16–O25. 24119076 10.1111/codi.12439

[11] Erlandsson J Lorinc E Ahlberg M . Tumour regression after radiotherapy for rectal cancer - Results from the randomised Stockholm III trial. Radiother Oncol 2019;135:178–86. 31015165 10.1016/j.radonc.2019.03.016

[12] Fokas E Strobel P Fietkau R . Tumor regression grading after preoperative chemoradiotherapy as a prognostic factor and individual-level surrogate for disease-free survival in rectal cancer. J Natl Cancer Inst 2017;109:djx095. 10.1093/jnci/djx095 29206996

[13] Kong JC Guerra GR Warrier SK . Prognostic value of tumour regression grade in locally advanced rectal cancer: a systematic review and meta-analysis. Colorectal Dis 2018;20:574–85. 29582537 10.1111/codi.14106

[14] Francois Y Nemoz CJ Baulieux J . Influence of the interval between preoperative radiation therapy and surgery on downstaging and on the rate of sphincter-sparing surgery for rectal cancer: the Lyon R90-01 randomized trial. J Clin Oncol 1999;17:2396. 10561302 10.1200/JCO.1999.17.8.2396

[15] Moore HG Gittleman AE Minsky BD . Rate of pathologic complete response with increased interval between preoperative combined modality therapy and rectal cancer resection. Dis Colon Rectum 2004;47:279–286. 14991488 10.1007/s10350-003-0062-1

[16] Petrelli F Sgroi G Sarti E . Increasing the interval between neoadjuvant chemoradiotherapy and surgery in rectal cancer: a meta-analysis of published studies. Ann Surg 2016;263:458–464. 24263329 10.1097/SLA.0000000000000368

[17] Gambacorta MA Masciocchi C Chiloiro G . Timing to achieve the highest rate of pCR after preoperative radiochemotherapy in rectal cancer: a pooled analysis of 3085 patients from 7 randomized trials. Radiother Oncol 2021;154:154–60. 32966845 10.1016/j.radonc.2020.09.026

[18] Huntington CR Boselli D Symanowski J . Optimal timing of surgical resection after radiation in locally advanced rectal adenocarcinoma: an analysis of the national cancer database. Ann Surg Oncol 2016;23:877–887. 26514119 10.1245/s10434-015-4927-z

[19] Sun Z Adam MA Kim J . Optimal timing to surgery after neoadjuvant chemoradiotherapy for locally advanced rectal cancer. J Am Coll Surg 2016;222:367–374. 26897480 10.1016/j.jamcollsurg.2015.12.017

[20] Agha R Abdall-Razak A Crossley E . STROCSS 2019 guideline: strengthening the reporting of cohort studies in surgery. Int J Surg 2019;72:156–65. 31704426 10.1016/j.ijsu.2019.11.002

[21] Ryan R Gibbons D Hyland JM . Pathological response following long-course neoadjuvant chemoradiotherapy for locally advanced rectal cancer. Histopathology 2005;47:141–146. 16045774 10.1111/j.1365-2559.2005.02176.x

[22] Cotti GC Pandini RV Braghiroli OFM . Outcomes of patients with local regrowth after nonoperative management of rectal cancer after neoadjuvant chemoradiotherapy. Dis Colon Rectum 2022;65:333–339. 34775415 10.1097/DCR.0000000000002197

[23] Cercek A Roxburgh CSD Strombom P . Adoption of total neoadjuvant therapy for locally advanced rectal cancer. JAMA Oncol 2018;4:e180071. 29566109 10.1001/jamaoncol.2018.0071 PMC5885165

[24] Dijkstra EA Nilsson PJ Hospers GAP . Locoregional failure during and after short-course radiotherapy followed by chemotherapy and surgery compared to long-course chemoradiotherapy and surgery - a five-year follow-up of the RAPIDO trial. Ann Surg 2023. Online ahead of print. 10.1097/SLA.0000000000005799 PMC10481913 36661037

[25] Jimenez-Fonseca P Salazar R Valenti V . Is short-course radiotherapy and total neoadjuvant therapy the new standard of care in locally advanced rectal cancer? A sensitivity analysis of the RAPIDO clinical trial. Ann Oncol 2022;33:786–93. 35462008 10.1016/j.annonc.2022.04.010

[26] Lefevre JH Mineur L Cachanado M . Does a longer waiting period after neoadjuvant radio-chemotherapy improve the oncological prognosis of rectal cancer?: three years’ follow-up results of the greccar-6 randomized multicenter trial. Ann Surg 2019;270:747–54. 31634178 10.1097/SLA.0000000000003530

[27] Lefevre JH Mineur L Kotti S . Effect of interval (7 or 11 weeks) between neoadjuvant radiochemotherapy and surgery on complete pathologic response in rectal cancer: a multicenter, randomized, controlled trial (GRECCAR-6). J Clin Oncol 2016;34:3773–80. 27432930 10.1200/JCO.2016.67.6049

[28] Van den Begin R Kleijnen JP Engels B . Tumor volume regression during preoperative chemoradiotherapy for rectal cancer: a prospective observational study with weekly MRI. Acta Oncol 2018;57:723–727. 29157069 10.1080/0284186X.2017.1400689

[29] Deidda S Elmore U Rosati R . Association of delayed surgery with oncologic long-term outcomes in patients with locally advanced rectal cancer not responding to preoperative chemoradiation. JAMA Surg 2021;156:1141–1149. 34586340 10.1001/jamasurg.2021.4566 PMC8482294

[30] Patel UB Taylor F Blomqvist L . Magnetic resonance imaging-detected tumor response for locally advanced rectal cancer predicts survival outcomes: MERCURY experience. J Clin Oncol 2011;29:3753–3760. 21876084 10.1200/JCO.2011.34.9068

[31] Sclafani F Brown G Cunningham D . PAN-EX: a pooled analysis of two trials of neoadjuvant chemotherapy followed by chemoradiotherapy in MRI-defined, locally advanced rectal cancer. Ann Oncol 2016;27:1557–1565. 27217542 10.1093/annonc/mdw215

[32] Sclafani F Brown G Cunningham D . Comparison between MRI and pathology in the assessment of tumour regression grade in rectal cancer. Br J Cancer 2017;117:1478–85. 28934761 10.1038/bjc.2017.320 PMC5680467

[33] Pang X Xie P Yu L . A new magnetic resonance imaging tumour response grading scheme for locally advanced rectal cancer. Br J Cancer 2022;127:268–77. 35388140 10.1038/s41416-022-01801-x PMC9296509

[34] Shaish H Aukerman A Vanguri R . Radiomics of MRI for pretreatment prediction of pathologic complete response, tumor regression grade, and neoadjuvant rectal score in patients with locally advanced rectal cancer undergoing neoadjuvant chemoradiation: an international multicenter study. Eur Radiol 2020;30:6263–73. 32500192 10.1007/s00330-020-06968-6

[35] Zhang XY Wang L Zhu HT . Predicting rectal cancer response to neoadjuvant chemoradiotherapy using deep learning of diffusion kurtosis MRI. Radiology 2020;296:56–64. 32315264 10.1148/radiol.2020190936

